# Comparison between repeatability, reproductive stage stratified repeatability, and relative risk models for prediction of breeding values for functional survival in rotationally crossbred sows

**DOI:** 10.1186/s12711-025-01019-4

**Published:** 2025-12-09

**Authors:** Bjarke G. Poulsen, Daniela Lourenco, Tage Ostersen, Bjarne Nielsen, Natália G. Leite, Mark Henryon, Ole F. Christensen

**Affiliations:** 1 Breeding & Genetics, Danish Agriculture and Food Council, Axelborg, Axeltorv 3, 1609 Copenhagen V, Denmark; 2 Center for Quantitative Genetics and Genomics, Aarhus University, C. F. Møllers Allé 3, 8000 Aarhus C, Denmark; 3 Department of Animal and Dairy Science, University of Georgia, Athens, GA USA

## Abstract

**Background:**

The aim of this study was to compare different statistical models for predicting breeding values for sow survival with right-censored phenotypes from rotationally crossbred and commercial sows. We tested two hypotheses. First, we hypothesized that single-trait relative risk models predict more accurate breeding values than single-trait linear repeatability models. Second, we hypothesized that a reproductive stage stratified linear repeatability model predicts more accurate breeding values than the standard single-trait linear repeatability models. The single-trait models predict breeding values for survival between farrowings, while the reproductive stage stratified models predict breeding values for both survival between a farrowing and the next service, and survival between a service and the next farrowing. The validation criterion was the Pearson correlation between adjusted phenotypes for the lifetime number of litters produced and predicted breeding values for survival converted to lifetime number of litters produced. All validation criteria were compared to one another and against zero using appropriate statistical tests and correction for multiple tests. Each model was constructed with two different multi-breed relationship matrices to ensure that the results were not affected by the choice between them.

**Results:**

The values of the validation criteria for the single-trait models were significantly larger than zero and similar (0.02). The values of the validation criteria for the reproductive stage stratified linear repeatability models were both significantly larger than zero and significantly larger than those from the single-trait models (0.04 vs. 0.02).

**Conclusions:**

The relative risk and linear repeatability single-trait models for survival between subsequent farrowings predicted equally accurate breeding values (0.02), while the linear repeatability two-trait models for survival from services to their subsequent farrowings and farrowings to the subsequent services predicted more accurate breeding values than the single-trait models (0.04 vs. 0.02). However, the accuracy of breeding values was small for all models because the survival phenotypes used for prediction were censored and the heritability of complete survival times was moderate (8–9%). Therefore, the comparison would benefit from reevaluation in other populations, and the models should be improved upon before implementation in practical breeding programs.

**Supplementary Information:**

The online version contains supplementary material available at 10.1186/s12711-025-01019-4.

## Background

Sow survival is an economically important trait for pork production systems. Sows are most profitable when they produce between six and nine litters throughout their lives [1–3]. Nevertheless, 62 percent of commercial sows produce fewer than six litters throughout their lives [4, 5]. Sow survival is also of societal importance because the absence of sow survivability indicates poor animal welfare [4, 6]. Improving sow survival will increase sow profitability and improve animal welfare.

Although sow survival is heritable [7, 8], several challenges make it difficult to generate much genetic progress for this trait. First, selection for sow survival should be based on data from commercial herds because the genetic correlation between sow survival traits across herd types is low (0.18–0.36) [9], and the commercial herds constitute the focal environment. However, breeding companies generally have limited access to commercial data. Second, analyses with commercial sows should use multibreed relationship matrices [10–13] because commercial sows are crossbred. However, these multibreed relationships have been scarcely used until recent years – likely because of limited availability of software tools to calculate the relationship matrices and being unfamiliar to researchers. Third, sow survival should be analyzed with statistical models that can combine information from sows that are dead (complete records) with information from sows that are alive (incomplete/right-censored records) [14], but there are several such statistical models [15–19] and the choice between them affects the genetic analysis. Lastly, sow survival is a complex trait that should likely be analyzed using a complex statistical model [4, 20], but using a statistical model with many parameters is not feasible for routine evaluation. Among these challenges, the first two can in principle be solved by using commercial data and multibreed relationship matrices. Therefore, in the following, we will focus on the challenges with statistical models for right-censored observations and with modeling the biological complexity of sow survival.

The motivation for the use of right-censored phenotypes is that complete phenotypes on sow survival often have little impact on the prediction of breeding values of selection candidates. This is because the complete phenotypes are available only when sows are culled, and such sows are separated by multiple generations from selection candidates. However, breeding values that are predicted with right-censored phenotypes are biased unless the statistical model accounts for this right-censoring [14].

Right-censored phenotypes can be accounted for with a statistical model for survival analysis [16, 17], such as a relative risk model [17]. However, the use of these models has been limited for genetic analysis because they are computationally demanding [21], while the additional computational costs may not lead to more accurate breeding values than the use of linear models [22, 23]. Instead, recent genetic analyses of sow survival have used linear repeatability models [7, 24]. However, it is not known whether linear repeatability models can predict as accurate breeding values for survival traits as relative risk models. On the one hand, many assumptions made in relative risk models may be more appropriate for survival data, while on the other hand, linear repeatability models may be robust to misspecification of the statistical model [17, 25]. Linear repeatability models could be more appropriate for modeling survival traits than relative risk models when survival rates are closer to 0.5 [17, 26], breeding values among animals of interest are predicted with similar amounts of information [22], and when the additive genetic variance on the underlying scale is small [22, 26]. For sow survival, the survival rate is about 0.88 [4, 5] and breeding values are not predicted with equal amounts of information, but the additive genetic variance on the underlying scale is small [7, 20, 26]. Thus, one of these characteristics is in favor of linear repeatability models while the other two are not. Therefore, it is not clear whether linear repeatability models are appropriate for sow survival.

The motivation for the use of complex models for sow survival is that it is a biologically complex trait. Sows can die from several causes, and the frequencies of these causes depend on the age of the sow and her stage in the reproductive cycle [4, 5]. A consequence of this is that genetic correlations between survival in different time periods in sows’ lives are less than one [20, 27]. Multi-trait genetic models can account for genetic correlations that are less than one. However, large multi-trait models for sow survival are generally not viable for routine prediction of breeding values in the industry because they are computationally demanding – e.g., when they include many fixed effects and (co)variance components. Multi-trait models for sow survival that regard survival in different parities as different traits would include many (co)variance components, because some sows surpass several parities [20, 24]. In contrast, multi-trait models for sow survival that regard survival in different stages of the reproductive cycle as different traits (e.g., gestation and lactation) will include fewer (co)variance components because there are only few such stages. To our knowledge, such multi-trait models have not been investigated previously. Therefore, it is of interest to determine whether breeding values for sow survival are more accurately predicted with single-trait models or with multi-trait models that stratify sows’ lives across stages in the reproductive cycle.

We tested two hypotheses in this study. First, we hypothesized that the breeding values for sow survival are more accurately predicted with a relative risk model than with a single-trait linear repeatability model. Second, we hypothesized that breeding values for sow survival are more accurately predicted with two-trait models stratified across reproductive stages than with single-trait models.

## Methods

### Overview of methods

**Fig. 1:**
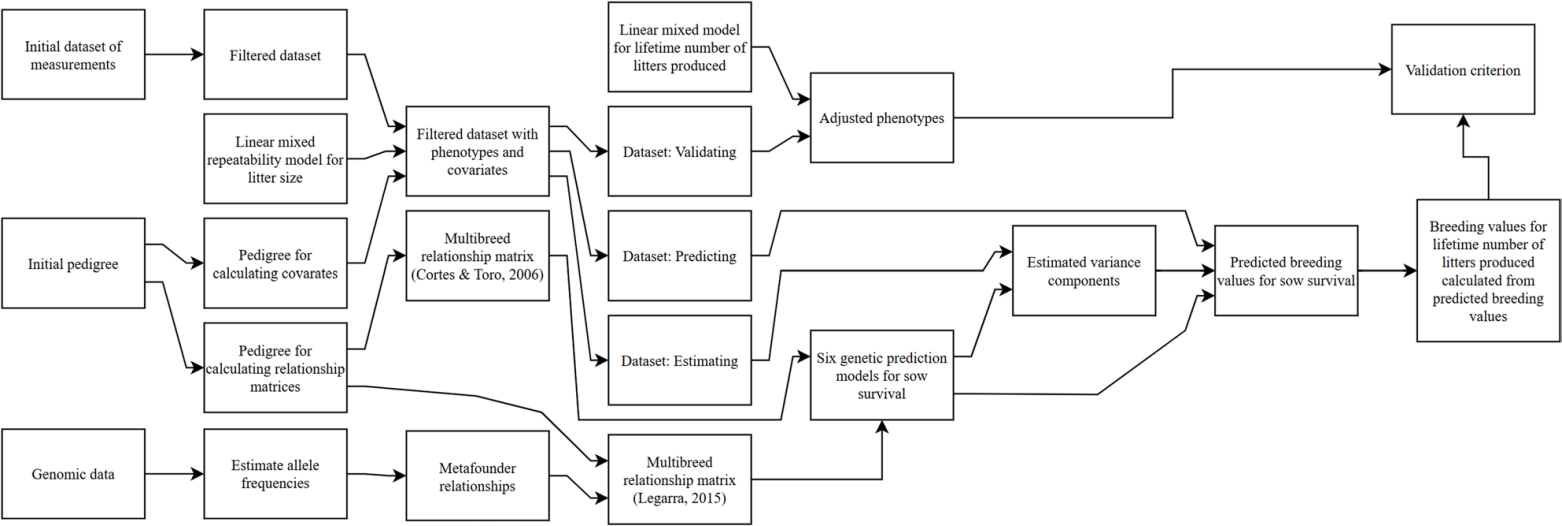
Overview over the methods used

We tested our two hypotheses by comparing predicted breeding values for the lifetime numbers of litters produced from six statistical models. Figure 1 provides a general overview of these models, which includes: two single-trait relative risk models fitted to farrowing-to-farrowing survival, two single-trait linear repeatability models fitted to farrowing-to-farrowing survival, and two two-trait linear repeatability models fitted to service-to-farrowing- and farrowing-to-service- survival. First, we constructed three datasets: one for estimating variance components, one for predicting breeding values, and one for validating the predicted breeding values. We then estimated variance components for each of the proposed statistical models using the dataset for estimating variance components. The resulting estimates of variance components were used in combination with their respective statistical model to predict breeding values for sow survival. The predicted breeding values for sow survival were then used to calculate the expected lifetime number of litters produced. Lastly, we calculated Pearson’s correlations between the expected lifetime number of litters produced and adjusted phenotypes for the lifetime number of litters produced among validation animals.

In the following sections, we first present the phenotype data and the performed quality control. Then, we present the phenotypes, covariates, and relationship matrices used in the analysis. Lastly, we present the statistical models and the calculation of the validation criterion for the predicted breeding values.

### Phenotype data

After the following quality control, the phenotype data for sow survival contained phenotypes from 223,518 gilts and sows in 89 Danish commercial herds. All animals with phenotypes were either F1 crossbreds or rotationally crossbred descendants from DanBred Landrace and DanBred Yorkshire. The phenotype data consisted of birth, service, and farrowing records. Quality controls were applied at herd and animal levels. In the phenotype data, the first birth was on January 1, 2014, and the last farrowing was on February 25, 2022. As explained in detail in the following section, to obtain this phenotype data, the initial phenotype data was treated as follows: (1) we omitted herds that mainly recorded data related to internal multiplication, were deviating from expected patterns in the management of the reproductive cycles of their animals, were only represented across few years in the dataset, and where the number of observations exhibited unexpected patterns across time; (2) we omitted animals with weak knowledge about the genetic links to the purebred populations, with low levels of heterosis (near-purebred animals), and animals that were serviced outside a reasonable window for age at first service; and (3) the remaining phenotype data was used to define three datasets used for different parts of the analysis.

#### Herd level quality control of phenotype data

Before the herd-level quality control, the phenotype data included 454 herds. The herd-level quality control of phenotype data was similar to that of Poulsen et al. [8]. Herds were evaluated based on records that were obtained between January 1, 2014, and February 25, 2022, on animals that were born between January 1, 2014, and December 31, 2017. Herds were kept for further analysis if more than 90% of their sows farrowed for the first time between 300 and 460 days of age, more than 97% of farrowing intervals were between 120 and 232 days, more than 90% of parity numbers were equal to expected parity numbers [8], more than 99% of sows’ genetic composition could be traced through the pedigree to purebred Landrace or Yorkshire animals, more than 80% of services were performed with semen from DanBred Duroc, and if they provided data for at least five consecutive years. We also omitted herds if they had large fluctuations in the number of observations across semesters or had irregular patterns for the number of observations using the following procedures: (1) we calculated the number of farrowings per combination of herd and semester (January–June or July–December), except for the last semester in the data because it was incomplete; (2) we divided these numbers of farrowings by the maximum number within herd to obtain ${f}_{herd,sem}$; and (3) we regarded herds as having large fluctuations or irregular patterns in the number of observations if any ${f}_{herd,sem}$ from before 2018 was more than 0.3 larger than the previous ${f}_{herd,sem}$, if any ${f}_{herd,sem}$ from before 2018 was more than 0.4 smaller than the previous ${f}_{herd,sem}$, or if any ${f}_{herd,sem}$ from 2018 or later was less than 0.7.

#### Sow-level quality control of phenotype data

Before the sow-level quality control, the phenotype data contained data from 282,804 animals that resided within the 89 herds that passed the herd-level quality control. Gilts and sows were kept for analysis if more than 99% of their genetic composition could be traced through the pedigree to purebred Landrace or Yorkshire animals, their pedigree-based level of heterosis was greater than 20%, and if they were serviced for the first time between 200 and 300 days of age.

#### Datasets used for analysis

**Fig. 2:**
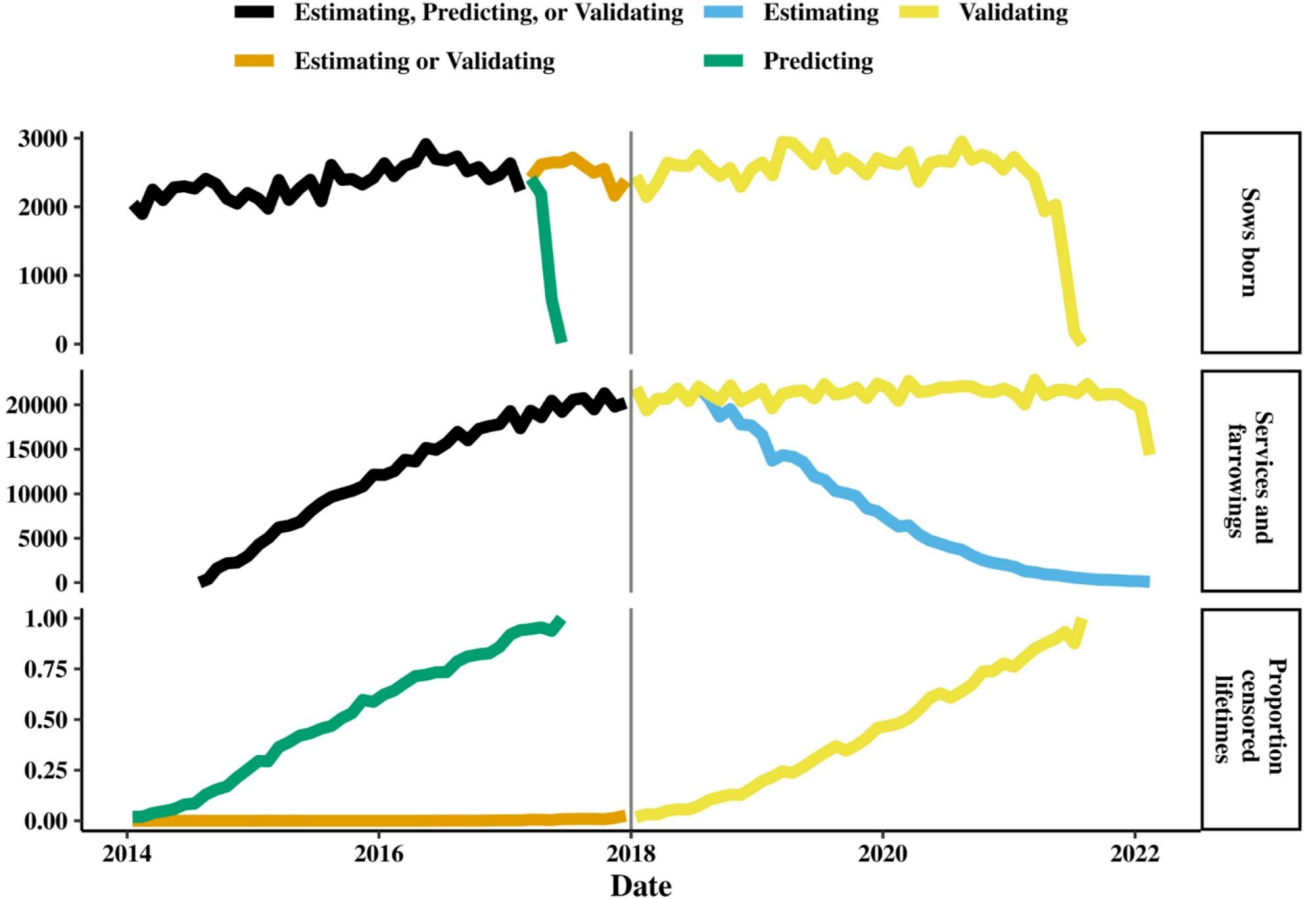
Amounts of sows born, services, and farrowings, and proportion of censored lifetimes across the datasets. Amounts per month are shown. Line colors denote one or more datasets. Proportion of censored lifetimes is the proportion of sows born at the date on the x-axis that was alive at the latest observation date in the dataset. The vertical grey line is the date used to split the Estimating dataset based on birthdates and the Predicting dataset based on observation dates

We used three datasets in this study: one dataset for estimating variance components (Estimating), one dataset for predicting breeding values (Predicting), and one dataset for validating the predicted breeding values (Validating). The datasets differed in which sows were included and how much information was available for each sow (Fig. 2). The Estimating dataset contained all information from sows that were born prior to January 1, 2018, and it contained mostly complete phenotypes for sow survival. The Predicting dataset contained information that was obtained prior to January 1, 2018, and it contained many right-censored phenotypes for sow survival. The Validating dataset consisted of all the available information (first birth: January 1, 2014; last farrowing; February 25, 2022), and it contained both complete and right-censored phenotypes for sow survival.

### Phenotypes

We used three survival phenotypes for predicting breeding values and one longevity phenotype for validating predicted breeding values (Table 1). The phenotype data did not contain culling dates. Instead, the phenotypes were constructed using the service and farrowing records based on the premises that service- or farrowing records are survival observations, and that prolonged absence of survival records indicates that sows have died [see Additional File 1 for the specific procedure].

**Table 1:** Descriptive statistics for the phenotypes across datasets

	Phenotype	Estimation	Prediction	Validation
Number of observations	FtoF	452,382	171,110	223,518
	StoF	486,399	244,331	
	FtoS	452,382	207,225	
	NoL	115,463		
Average ± standard deviation	FtoF	0.23 ± 0.42	0.17 ± 0.38	3.22 ± 2.25 (14)
	StoF	0.07 ± 0.25	0.07 ± 0.25	
	FtoS	0.17 ± 0.38	0.12 ± 0.33	
	NoL^a^	3.96 ± 2.33 (14)		

^a^Cells in this row also have maxima in parentheses. Estimation: dataset for estimating variance components. Prediction: dataset for predicting breeding values. Validation: dataset for validating breeding values. FtoF: binary phenotype for survival between a farrowing and the following farrowing. StoF: binary phenotype for survival between a service and the following farrowing. FtoS: binary phenotype for survival between a farrowing and the following service. NoL: Lifetime number of litters produced

**Fig. 3:**
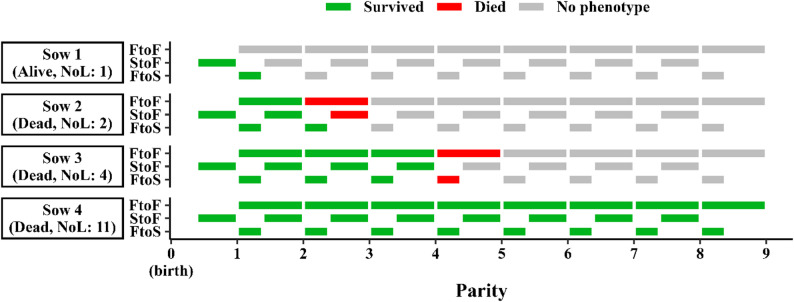
Relationship between phenotypes for four hypothetical sows. NoL: Lifetime number of litters produced. FtoF: Survival between two successive farrowings. StoF: Survival between a service and the subsequent farrowing. FtoS: Survival between a farrowing and the subsequent service. Colored boxes: Time periods where the sows could obtain a phenotype for each trait. Note that time periods for FtoF, StoF, and FtoS are nested within parity number

The survival phenotypes were *Survival from a farrowing to the next farrowing* (FtoF), *Survival from a service to the following farrowing* (StoF), and *Survival from a farrowing to the following service* (FtoS). Common to all of these phenotypes was that the measurements were binary (death = 1 and survival = 0), that each sow could have up to eight repeated measurements of the same phenotype (Fig. 3), and that only one measurement could be ‘1’ per sow (sows can only die once). Repeated measurements are referred to as *time periods* that are numbered based on the number of parities the sow had produced prior to that time period – i.e., time periods for FtoF and FtoS were numbered from one to eight, and time periods for StoF were numbered from zero to seven. The longevity phenotype was the *lifetime number of litters produced* (NoL) from the first service to death.

### Covariates

We calculated covariates for breed percentage, heterosis, and reproductive performance as in Poulsen et al. [8]. The covariates for breed percentage and heterosis were calculated using pedigree information and the infinitesimal model [8], while the reproductive performance of a sow was defined as its predicted permanent random effect from a mixed linear model for litter size [9] [see Additional File 2 for the model specifics].

### Pedigree data

We used two pedigrees in this study. The first pedigree was used to calculate covariates for breed percentage and heterosis, while the second pedigree was used to calculate relationship matrices. We used two pedigrees, because pedigree depth is more important for the calculation of breed percentage and heterosis than for the prediction of breeding values. Both pedigrees contained Landrace, Yorkshire, and crossbred animals. The pedigree for calculating covariates contained animals with phenotypes and their ancestors traced back 10 generations. The pedigree for calculating relationship matrices contained animals with phenotypes and their ancestors within the previous five generations.

### Genotype data

The genotype data was only used to estimate the metafounder relationships for Landrace and Yorkshire [11]. It consisted of imputed genotypes from routine evaluations in the DanBred breeding program and animals within the pedigree that were used to calculate relationship matrices. Initially, 44,633 markers were available for Landrace and Yorkshire combined (Landrace: 38,891; Yorkshire: 38,361; shared: 32,619). However, only the markers that were shared across the two populations were kept for further analysis. Quality control for marker genotypes was performed as part of the commercial breeding program. In total, 18,113 animals had genotype information (Landrace: 8677; Yorkshire: 9436).

### Multibreed relationship matrices

We used two types of multibreed relationship matrices that have been recommended for analyzing phenotypes from rotationally crossbred animals [12, 13] but may predict different breeding values [28]. The first type consisted of the partial relationship matrices proposed by García-Cortés and Toro (GT) [10]. The second type was the relationship matrix with metafounders (MF) [11].

#### The partial relationship matrices proposed by García-Cortés and Toro

These relationship matrices were developed specifically for genetic analyses of crossbred animals [10, 12]. In this study, the GT relationship matrices consisted of three partial relationship matrices: the partial relationship matrix for the vector of breed-specific effects from Landrace, ${\mathbf{A}}_{\text{L}}$; the partial relationship matrix for the vector of breed-specific effects from Yorkshire, ${\mathbf{A}}_{\text{Y}}$; and the partial relationship matrix for the vector of segregation effects between Landrace and Yorkshire, ${\mathbf{A}}_{\text{S}}$.

#### The relationship matrix with metafounders

The MF relationship matrix was not specifically developed for genetic analyses of rotationally crossbred animals. Nevertheless, it is similar to the GT relationship matrices under certain conditions [11, 13]. With the MF relationship parametrization, additive genetic effects are modeled with a single relationship matrix, ${\mathbf{A}}_{\text{MF}}$. For this study, the MF relationship matrix was calculated following [11] by: (1) declaring one metafounder per purebred population; (2) calculating the metafounder relationships using estimated allele frequencies in the base populations [29, 30], $${\varvec{\Gamma}}=\left[\begin{array}{cc}{\gamma }_{L}& {\gamma }_{LY}\\ {\gamma }_{YL}& {\gamma }_{Y}\end{array}\right]=\left[\begin{array}{cc}0.65& 0.09\\ 0.09& 0.55\end{array}\right]$$, and (3) calculating the MF relationship matrix [11].

The MF relationship matrix requires that all parents of base animals are assigned to a metafounder. However, in this study, some of the base animals were rotationally crossbred (N = 330). As a solution, we added a unique F1 dummy parent to rotationally crossbred base animals with a missing mother (Legarra A, personal communication in email, June 8, 2022). The sires of dummy parents were assumed to descend from the Landrace metafounder, while the dams of dummy parents were assumed to descend from the Yorkshire metafounder.

### Statistical models for predicting breeding values

#### Single-trait relative risk models

We analyzed FtoF using two relative risk models [17] with different relationship matrices. The general structure of these models was:

1 $$\begin{array}{c}{\rm E}\left(\mathbf{y}\right)=exp\left(\mathbf{X}\mathbf{b}+\mathbf{W}\mathbf{u}+\mathbf{Z}\mathbf{a}\right),\end{array}$$

where **y** is a vector of phenotypes for FtoF; **b** is a vector of fixed effects for time period, herd-year combination, and month at the start of the time period, and regression coefficients nested within time periods for breed percentage of Yorkshire, heterosis, age at first service, age at first service squared, and litter size in the previous parity; **u** is a vector of random effects for herd-year-quarter at the start of the time period, which was included to capture random herd-specific seasonal fluctuations from the broader herd-year and month fixed effects; **a** is a vector of additive genetic effects; and **X**, **W**, and **Z** are design matrices.

The two relative risk models were defined using Eq. (1) and the following distributions of random effects:

2 $$\begin{aligned} & Single\_Relative\_GT: \\ & \quad \left[\begin{array}{c}\mathbf{u}\\ \mathbf{a}\end{array}\right]\sim N\left( \begin{array}{cc}\left[\begin{array}{c}\mathbf{0}\\ \mathbf{0}\end{array}\right],& \left[\begin{array}{cc}{\sigma }_{U}^{2}\mathbf{I}& \mathbf{0}\\ \mathbf{0}& {\sigma }_{{A}_{L}}^{2}{\mathbf{A}}_{L}+{\sigma }_{{A}_{Y}}^{2}{\mathbf{A}}_{Y}\end{array}\right]\end{array}\right),\end{aligned}$$

3 $$\begin{aligned} & Single\_Relative\_MF:\\ & \quad \left[\begin{array}{c}\mathbf{u}\\ \mathbf{a}\end{array}\right]\sim N\left( \begin{array}{cc}\left[\begin{array}{c}\mathbf{0}\\ \mathbf{0}\end{array}\right],& \left[\begin{array}{cc}{\sigma }_{U}^{2}\mathbf{I}& \mathbf{0}\\ \mathbf{0}& {\sigma }_{{A}_{MF}}^{2}{\mathbf{A}}_{MF}\end{array}\right]\end{array}\right),\end{aligned}$$

where **u** and **a** are as defined for Eq. (1); ${\sigma }_{U}^{2}$ is the variance of herd-year-quarter effects; ${\sigma }_{{A}_{L}}^{2}$ is the variance of breed-specific effects from Landrace; ${\sigma }_{{A}_{Y}}^{2}$ is the variance of breed-specific effects from Yorkshire; ${\sigma }_{{A}_{MF}}^{2}$ is the additive genetic variance in the ancestral population; **0** is a vector or matrix of zeros; **I** is an identity matrix; and ${\mathbf{A}}_{L}$, ${\mathbf{A}}_{Y}$, and ${\mathbf{A}}_{MF}$ are the relationship matrices defined in the previous section.

The relative risk models were defined as sire-dam models because preliminary analyses with animal models provided unreasonable estimates of variance components (e.g., ${\sigma }_{{A}_{L}}^{2}>1000$). For the same reason, we did not include a breed segregation term (covariance ${\sigma }_{{A}_{S}}^{2}{\mathbf{A}}_{S}$) in the Single_Relative_GT model (Eq. 2).

The parameters in the relative risk models were estimated using a generalized linear mixed model with the logarithm link function and the Poisson variance function with a dispersion parameter [17]. We used the Poisson variance function rather than the theoretically appropriate Binomial variance function because models with the two variance functions provide similar estimates and the computational times are smaller for models with the Poisson variance function [17].

#### Single-trait linear repeatability models

We also analyzed FtoF using two linear repeatability animal models. The general structure of these models was:

4 $$\begin{array}{c}\mathbf{y}=\mathbf{Xb}+\mathbf{Wu}+\mathbf{Za}+\mathbf{e},\end{array}$$

where **e** is a vector of residuals, and the other terms are as defined for the relative risk models (Eq. 1).

The two single-trait linear repeatability animal models were defined using Eq. (4) and the following distributions of random effects:

5 $$\begin{aligned} & Single\_Linear\_GT:\left[ {\begin{array}{*{20}c} {\mathbf{u}} \\ {\mathbf{a}} \\ {\mathbf{e}} \\ \end{array} } \right]\sim N\left( {\left[ {\begin{array}{*{20}c} \mathbf{0} \\ \mathbf{0} \\ \mathbf{0} \\ \end{array} } \right],} \right. \\ & \quad \left. {\left[ {\begin{array}{*{20}c} {\sigma _{U}^{2} {\mathbf{I}}} & {} & {symm.} \\ \mathbf{0} & {\sigma _{{A_{L} }}^{2} {\mathbf{A}}_{L} + \sigma _{{A_{Y} }}^{2} {\mathbf{A}}_{Y} + \sigma _{{A_{S} }}^{2} {\mathbf{A}}_{S} } & {} \\ \mathbf{0} & \mathbf{0} & {\sigma _{E}^{2} {\mathbf{I}}} \\ \end{array} } \right]} \right), \\ \end{aligned} $$

6 $$\begin{array}{*{20}c} \begin{aligned} & Single\_Linear\_MF: \\ & \quad \left[ {\begin{array}{*{20}c} {\mathbf{u}} \\ {\mathbf{a}} \\ {\mathbf{e}} \\ \end{array} } \right]\sim N\left( {\begin{array}{*{20}c} {\left[ {\begin{array}{*{20}c} \mathbf{0} \\ \mathbf{0} \\ \mathbf{0} \\ \end{array} } \right],} & {\left[ {\begin{array}{*{20}c} {\sigma _{U}^{2} {\mathbf{I}}} & {} & {symm.} \\ \mathbf{0} & {\sigma _{{A_{{MF}} }}^{2} {\mathbf{A}}_{{MF}} } & {} \\ \mathbf{0} & \mathbf{0} & {\sigma _{E}^{2} {\mathbf{I}}} \\ \end{array} } \right]} \\ \end{array} } \right), \\ \end{aligned} \\ \end{array} $$

where ${\sigma }_{{A}_{S}}^{2}$ is the additive genetic segregation variance; ${\mathbf{A}}_{S}$ is the partial relationship matrix for segregation effects between Landrace and Yorkshire, and the other terms are as defined for the distributions of random effects in the relative risk models Eqs. (2 and 3).

#### Two-trait linear repeatability models

StoF and FtoS were simultaneously analyzed with two-trait linear repeatability animal models. The general structure of these models was:

7 $$\begin{array}{c}\left[\begin{array}{c}{\mathbf{y}}_{StoF}\\ {\mathbf{y}}_{FtoS}\end{array}\right]=\mathbf{Xb}+\mathbf{Wu}+\mathbf{Za}+\mathbf{e},\end{array}$$

where ${\mathbf{y}}_{StoF}$ is a vector of phenotypes for StoF; ${\mathbf{y}}_{FtoS}$ is a vector of phenotypes for FtoS; **b** is a vector of fixed effects for time period, herd-year combination, and month at the start of the time period, and regression coefficients nested within time periods for breed percentage of Yorkshire, heterosis, age at first service, age at first service squared, and litter size in the parity prior to the time period (omitted for StoF in the first time period); and **u**, **a**, **e**, **X**, **W**, and **Z** are as defined for Eq. (4). In Eq. (7), both fixed and random effects were nested within trait.

The two two-trait linear repeatability animal models were defined using Eq. (7) and the following distributions of random effects:

8 $$\begin{aligned} & Two\_Linear\_GT:\left[ {\begin{array}{*{20}c} {\mathbf{u}} \\ {\mathbf{a}} \\ {\mathbf{e}} \\ \end{array} } \right]\sim N\left( {\left[ {\begin{array}{*{20}c} \mathbf{0} \\ \mathbf{0} \\ \mathbf{0} \\ \end{array} } \right]} \right., \\ & \quad \left. {\left[ {\begin{array}{*{20}c} {\varvec{\Sigma}_{U} \otimes {\mathbf{I}}} & {} & {symm.} \\ \mathbf{0} & {\varvec{\Sigma}_{{A_{L} }} \otimes {\mathbf{A}}_{L} + \varvec{\Sigma}_{{A_{Y} }} \otimes {\mathbf{A}}_{Y} + \varvec{\Sigma}_{{A_{S} }} \otimes {\mathbf{A}}_{S} } & {} \\ \mathbf{0} & \mathbf{0} & {\varvec{\Sigma}_{E} \otimes {\mathbf{I}}} \\ \end{array} } \right]} \right), \\ \end{aligned} $$

9 $$\begin{aligned} & Two\_Linear\_MF:\left[ {\begin{array}{*{20}c} {\mathbf{u}} \\ {\mathbf{a}} \\ {\mathbf{e}} \\ \end{array} } \right]\sim N\left( {\left[ {\begin{array}{*{20}c} \mathbf{0} \\ \mathbf{0} \\ \mathbf{0} \\ \end{array} } \right],} \right. \\ & \quad \left. { \left[ {\begin{array}{*{20}c} {\varvec{\Sigma}_{U} \otimes {\mathbf{I}}} & {} & {symm.} \\ \mathbf{0} & {\varvec{\Sigma}_{{A_{{MF}} }} \otimes {\mathbf{A}}_{{MF}} } & {} \\ \mathbf{0} & \mathbf{0} & {\varvec{\Sigma}_{E} \otimes {\mathbf{I}}} \\ \end{array} } \right]} \right) \\ \end{aligned} $$

where ${{\varvec{\Sigma}}}_{U}$ is a 2 × 2 diagonal covariance matrix for herd-year-quarter effects; ${{\varvec{\Sigma}}}_{{A}_{MF}}$ is a 2 × 2 covariance matrix for additive genetic effects with parameters that refer to the ancestral population; ${{\varvec{\Sigma}}}_{{A}_{L}}$ is a 2 × 2 covariance matrix for breed-specific effects from Landrace; ${{\varvec{\Sigma}}}_{{A}_{Y}}$ is a 2 × 2 covariance matrix for breed-specific effects from Yorkshire; ${{\varvec{\Sigma}}}_{{A}_{S}}$ is a 2 × 2 covariance matrix for segregation effects between Landrace and Yorkshire; ${{\varvec{\Sigma}}}_{E}$ is a diagonal 2 × 2 covariance matrix for residual effects, and the remaining parameters are as previously defined Eqs. (5) and (6).

#### Permanent environmental effects

The statistical models for predicting breeding values are repeatability models because sows have phenotypes for all the time periods they could have died in. Generally, repeatability models should include a permanent environmental effect. However, for analysis of survival phenotypes, the variance parameter for such an effect is both difficult to estimate and interpret, because of the phenotype structure that animals only can die once and that they must be alive at all previous time periods before they die [7, 31]. This is further shown by our inability to estimate this variance parameter during preliminary analyses. Therefore, none of the prediction models included a permanent environmental effect.

#### Heritabilities

We calculated two heritabilities for each of the six prediction models, one for Landrace and one for Yorkshire. For the linear models, the heritabilities were defined as ${h}_{L}^{2}=\frac{{\sigma }_{{A}_{L}}^{2}}{{\sigma }_{{A}_{L}}^{2}+{\sigma }_{U}^{2}+{\sigma }_{E}^{2}}$ for Landrace and ${h}_{Y}^{2}=\frac{{\sigma }_{{A}_{Y}}^{2}}{{\sigma }_{{A}_{Y}}^{2}+{\sigma }_{U}^{2}+{\sigma }_{E}^{2}}$ for Yorkshire. The models with the MF relationship matrices don’t directly provide breed-specific genetic variances. Instead, these were calculated as ${\sigma }_{{A}_{L}}^{2}={\sigma }_{{A}_{MF}}^{2}\left(1-\frac{1}{2}{\gamma }_{L}\right)$ for Landrace and ${\sigma }_{{A}_{Y}}^{2}={\sigma }_{{A}_{MF}}^{2}\left(1-\frac{1}{2}{\gamma }_{Y}\right)$ for Yorkshire [11], where ${\sigma }_{{A}_{MF}}^{2}$ is an additive genetic variance in the ancestral population, and ${\gamma }_{L}$ and ${\gamma }_{Y}$ are metafounder relationships. For the relative risk models, the heritabilities are time-dependent ${h}_{L}^{2}\left(t\right)\approx \frac{{\sigma }_{{A}_{L}}^{2}}{{\sigma }_{{A}_{L}}^{2}+{\sigma }_{U}^{2}+\phi {\left(\Delta \left(\text{t}\right)\right)}^{-1}}$ for Landrace and ${h}_{Y}^{2}\left(t\right)\approx \frac{{\sigma }_{{A}_{Y}}^{2}}{{\sigma }_{{A}_{Y}}^{2}+{\sigma }_{U}^{2}+\phi {\left(\Delta \left(\text{t}\right)\right)}^{-1}}$ for Yorkshire [17], where $\phi$ is the dispersion parameter, and $\Delta \left(\text{t}\right)$ is related to the proportion of animals alive after time $\text{t}$. We used $\Delta \left(\text{t}\right)=1$ for simplicity because heritabilities were not the focus of this study.

### Validation

It is not straightforward to validate predicted breeding values for sow survival. First, sow survival is a repeated trait for the prediction models used in this study with more repeated observations for the phenotypically best animals. Second, predicted breeding values for sow survival should not be validated with only one time period because the genetic correlation for survival across different time periods is less than one [20]. Lastly, predicted breeding values from the two-trait models and the single-trait models cannot be directly compared because they split the lives of sows into different time periods (FtoF vs. StoF and FtoS). Therefore, instead of validating the predicted breeding values against the phenotype definitions that they were predicted with, we transformed all predicted breeding values to the longevity trait, NoL, and validated them based on their ability to predict adjusted phenotypes for NoL. This approach: (1) enabled us to compare predicted breeding values from the two-trait and the single-trait models on the same scale, (2) has greater statistical power for a test of the difference between correlations [32] because the heritability is larger for longevity traits than for survival traits [7, 9, 20], and (3) may have an expected regression coefficient of adjusted phenotypes onto transformed predicted breeding values equal to one [see Additional Files 3]. The ability to transform between breeding values for survival and longevity was checked using a small analysis of simulated data [see Additional Files 3].

#### Calculating predicted breeding values for lifetime number of litters produced from predicted breeding values for survival

We calculated the predicted breeding values for NoL, ${\widehat{\mathbf{a}}}_{NoL}$, differently for the three types of prediction models: single-trait relative risk, single-trait linear, and multi-trait linear. However, all three approaches are based on the expectation formula [33]:

10 $$\begin{array}{c}{\widehat{\mathbf{a}}}_{NoL}=E\left({{\varvec{y}}}_{NoL}|\widehat{\mathbf{a}}\right)=\sum_{x=0}^{n}xf\left(x,\widehat{\mathbf{a}}\right),\end{array}$$

where $x$ is the number of litters produced, ${\widehat{\mathbf{a}}}_{NoL}$ is a vector of expected lifetime numbers of litters produced, $n=9$ is the maximum number of parities a sow could achieve based on the prediction models, $f\left(x,\widehat{\mathbf{a}}\right)$ is a vector of probabilities for sows dying after producing exactly $x$ litters. The vector $f\left(x,\widehat{\mathbf{a}}\right)$ was calculated using conditional probabilities of survival during the current and previous time periods [33] as:

11 $$\begin{array}{c}f\left(x,\widehat{\mathbf{a}}\right)=\left(1-p\left(x,\widehat{\mathbf{a}}\right)\right)\prod_{y=0}^{x-1}p\left(y,\widehat{\mathbf{a}}\right),\end{array}$$

where $p\left(x,\widehat{\mathbf{a}}\right)$ is the vector of probabilities of survival between farrowings $x$ and $x+1$, given survival until farrowing $x$; $p\left(y,\widehat{\mathbf{a}}\right)$ is the vector probabilities of survival between farrowings $y$ and $y+1$, given survival until farrowing $y$; $p\left(-1,\widehat{\mathbf{a}}\right)=1$ because it is the probability that sows are alive when we start observing them; and products between vectors are elementwise. The vectors of conditional probabilities of survival, $p\left(x,\widehat{\mathbf{a}}\right)$ and $p\left(y,\widehat{\mathbf{a}}\right)$, can be calculated for all the prediction models. However, the equations for doing so differ between the prediction models. In the following, we only show the calculation of $p\left(x,\widehat{\mathbf{a}}\right)$ because $p\left(x,\widehat{\mathbf{a}}\right)=p\left(y,\widehat{\mathbf{a}}\right)$ when $x=y$.

For the Single_Relative_GT and Single_Relative_MF models, $p\left(x,\widehat{\mathbf{a}}\right)$ was calculated as:

12 $$\begin{array}{c}1-p\left(x,\widehat{\mathbf{a}}\right)=\left\{\begin{array}{c}\mathbf{0}, x=0\\ \text{exp}\left(1\mu +{\widehat{\mathbf{a}}}_{FtoF}\right), x\in \left\{\text{1,2},3,\dots , 8\right\}\\ \mathbf{1}, otherwise\end{array}\right.,\end{array}$$

where ${\widehat{\mathbf{a}}}_{FtoF}$ is a vector of predicted breeding values for FtoF, $\mathbf{1}$ is a vector of ones, and $\mu$ is the average effect size among validation animals for mortality between farrowings $x$ and $x+1$. The average effect size, $\mu$, was calculated as $\mu =\text{log}\left(1-{p}_{FtoF}\left(x\right)\right)$ based on $E\left(1-p\left(x,\widehat{\mathbf{a}}\right)\right)=1-1{p}_{FtoF}\left(x\right)=\text{exp}\left(1\mu \right)$, where ${p}_{FtoF}\left(x\right)$ is the average survival rate among validation animals between farrowings $x$ and $x+1$. The vector of predicted breeding values, $\widehat{\mathbf{a}}$, was centered because ${p}_{FtoF}\left(x\right)$ is affected by the average breeding value among validation animals. Lastly, we assumed that sows could not die before the first farrowing – i.e., we assumed that $p\left(0,\widehat{\mathbf{a}}\right)=\mathbf{1}$, where $\mathbf{1}$ is a vector of ones.            

For Single_Linear_GT and Single_Linear_MF, $p\left(x,\widehat{\mathbf{a}}\right)$ was calculated as:

13 $$\begin{array}{c}p\left(x,\widehat{\mathbf{a}}\right)=\left\{\begin{array}{c}1, x=0\\ {p}_{FtoF}\left(x\right)1-{\widehat{\mathbf{a}}}_{FtoF}, x\in \left\{\text{1,2},3,\dots , 8\right\}\\ 0, otherwise\end{array}\right.,\end{array}$$

where all terms are defined as for Single_Relative_GT and Single_Relative_MF (Eq. 12).

For the Two_Linear_GT and Two_Linear_MF, $p\left(x,\widehat{{\varvec{a}}}\right)$ was calculated as:

14 $$\begin{aligned} & p\left(x,\widehat{\mathbf{a}}\right)=\\ & \quad \left\{\begin{array}{c}{p}_{StoF}\left(x\right)\mathbf{1}-{\widehat{\mathbf{a}}}_{StoF}, x=0\\ \left({p}_{FtoS}\left(x\right)\mathbf{1}-{\widehat{\mathbf{a}}}_{FtoS}\right)\left({p}_{StoF}\left(x\right)\mathbf{1}-{\widehat{{\varvec{a}}}}_{StoF}\right), \\ \qquad \qquad \qquad x\in \left\{\text{1,2},3,\dots , 7\right\}\\ {p}_{FtoS}\left(x\right)\mathbf{1}-{\widehat{\mathbf{a}}}_{FtoS}, x=8\\ \mathbf{0}, otherwise\end{array}\right.\end{aligned}$$

where ${p}_{FtoS}\left(x\right)$ is the average rate of survival among validation animals during time period $x$ for FtoS, ${p}_{StoF}\left(x\right)$ is the average rate of survival among validation animals during time period $x$ for StoF, ${\widehat{\mathbf{a}}}_{FtoS}$ is a vector of predicted breeding values for FtoS, ${\widehat{\mathbf{a}}}_{StoF}$ is a vector of predicted breeding values for StoF, and products between vectors are elementwise. Equation (14) contains four cases because ${p}_{FtoS}\left(0\right)$ is not defined and because ${p}_{StoF}\left(8\right)$ was not calculated. The vectors of predicted breeding values for FtoS and StoF were centered such that their averages among validation animals were equal to zero.

#### Adjusted phenotypes for lifetime number of litters produced

Phenotypes from validation animals were adjusted using estimates of fixed effects from a linear mixed model for NoL as:

15 $$\begin{array}{c}{\mathbf{y}}_{A}={\mathbf{y}}_{\text{NoL}}-X\widehat{\mathbf{b}},\end{array}$$

where ${\mathbf{y}}_{A}$ is a vector of adjusted phenotypes for NoL, ${\mathbf{y}}_{\text{NoL}}$ is a vector of phenotypes for NoL, $\widehat{\mathbf{b}}$ is a vector of estimated fixed effects and $\mathbf{X}$ is the design matrix for fixed effects. The elements, ${\mathbf{y}}_{\text{NoL}}$, $\widehat{\mathbf{b}}$**,** and $\mathbf{X}$, in Eq. (15) originated from the following mixed model for NoL in the Validation dataset:

16 $$\begin{array}{c}{\mathbf{y}}_{\text{NoL}}=\mathbf{Xb}+\mathbf{Wu}+\mathbf{e},\end{array}$$

where ${\mathbf{y}}_{\text{NoL}}$ is a vector of phenotypes for NoL; **b** is a vector of fixed effects for herd-year combination at first service, the month at first service, and regression coefficients for breed percentage of Yorkshire, heterosis, age at first service, age at first service squared, and reproductive performance; **u** is a vector of random effects for herd-year-quarter at first service; **e** is a vector of residuals; and **X** and $\mathbf{W}$ are design matrices. The random effects in this statistical model were assumed to be distributed as:

17 $$\begin{array}{c}\left[\begin{array}{c}\mathbf{u}\\ \mathbf{e}\end{array}\right]\sim N\left( \begin{array}{cc}\left[\begin{array}{c}\mathbf{0}\\ \mathbf{0}\end{array}\right],& \left[\begin{array}{cc}{\sigma }_{U}^{2}\mathbf{I}& \mathbf{0}\\ \mathbf{0}& {\sigma }_{E}^{2}\mathbf{I}\end{array}\right]\end{array}\right),\end{array}$$

where all parameters are as defined for the distribution of random effects in Eq. (5).

#### Validation criterion

Our goal was to determine which model predicted the most accurate breeding values for lifetime survival. However, true breeding values are not observed. Instead, our validation criterion was the predicted breeding values’ ability to predict adjusted phenotypes, $cor\left({{\varvec{y}}}_{A},\widehat{{\varvec{a}}}\right)$, where $cor\left(\dots \right)$ is Pearson’s correlation coefficient, ${{\varvec{y}}}_{A}$ is a vector of adjusted phenotypes (Eq. 15), and $\widehat{{\varvec{a}}}$ is a vector of predicted breeding values. We calculated one validation criterion value for each prediction model. The validation criteria were calculated using adjusted phenotypes from the sows in the Validating dataset that were serviced for the first time in year 2018. 

We tested whether the validation criteria were larger than zero using *t*-tests and whether they were different from one another using the Hotelling–Williams T-squared test [32, 34, 35]. This resulted in 6 individual tests against zero and 15 pair-wise tests between validation criteria. Therefore, we used the Bonferroni correction to account for multiple testing with 21 tests. We also report the accuracy of predicted breeding values, computed as ${\sqrt{{h}_{{{\varvec{y}}}_{A}}^{2}}}^{-1}cor\left({{\varvec{y}}}_{A},\widehat{{\varvec{a}}}\right)$, because it has a more intuitive interpretation than the original validation criterion.

#### Dispersion coefficient

For multi-trait selection, it is important that the scaling of predicted breeding values is accurate across traits. Therefore, we also report estimates of the dispersion coefficient for the predicted breeding values, $\upbeta$, which was obtained with the following linear model:

18 $$\begin{array}{c}{{\mathbf{y}}}_{A}=\mu 1+\beta \widehat{{\mathbf{a}}}+\mathbf{e},\end{array}$$

where ${{\mathbf{y}}}_{A}$ is a vector of adjusted phenotypes for NoL, $\mu$ is an intercept, $\mathbf{1}$ is a vector of ones, $\upbeta$ is a regression coefficient, $\widehat{{\mathbf{a}}}$ is a vector of predicted breeding values, and $\mathbf{e}$ is a vector of residuals. The expected value of $\upbeta$ should be 1 [see Additional File 3]. The dispersion coefficients were not subject to statistical tests.

#### Heritability of adjusted phenotypes for lifetime number of litters produced

The heritability for NoL was used to calculate the accuracy of predicted breeding values. The heritability was calculated with the estimated variance components from the following linear animal model (NoL_GT):

19 $$\begin{array}{c}\mathbf{y}=\mathbf{Xb}+\mathbf{Wu}+\mathbf{Za}+\mathbf{e},\end{array}$$

where **y** is a vector of phenotypes for NoL from the Estimating dataset; **b** is a vector of fixed effects for herd-year combination at first service, the birth month at first service, and regression coefficients for breed percentage of Yorkshire, heterosis, age at first service, age at first service squared, and reproductive performance; **u** is a vector of effects for herd-year-quarter at first service; **a** is a vector of additive genetic effects; **e** is a vector of residuals; and **X**, **W**, and **Z** are design matrices. The random effects were assumed to be distributed as:

20 $$\begin{aligned} & \left[ {\begin{array}{*{20}c} {\mathbf{u}} \\ {\mathbf{a}} \\ {\mathbf{e}} \\ \end{array} } \right]\sim N\left( {\left[ {\begin{array}{*{20}c} \mathbf{0} \\ \mathbf{0} \\ \mathbf{0} \\ \end{array} } \right],} \right. \\ & \quad \left. {\left[ {\begin{array}{*{20}c} {\sigma _{U}^{2} {\mathbf{I}}} & {} & {symm.} \\ \mathbf{0} & {\sigma _{{A_{L} }}^{2} {\mathbf{A}}_{L} + \sigma _{{A_{Y} }}^{2} {\mathbf{A}}_{Y} } & {} \\ \mathbf{0} & \mathbf{0} & {\sigma _{E}^{2} {\mathbf{I}}} \\ \end{array} } \right]} \right), \\ \end{aligned} $$

where all parameters are as defined as for the distribution of random effects in Eq. (5). We did not include a segregation term in NoL_GT because preliminary analyses showed that this effect shared much information with the residual. The heritability of NoL was then calculated from the estimated variance components as ${h}_{{{\mathbf{y}}}_{A}}^{2}=\frac{\frac{1}{2}\left({\sigma }_{{A}_{L}}^{2}+{\sigma }_{{A}_{Y}}^{2}\right)}{\frac{1}{2}\left({\sigma }_{{A}_{L}}^{2}+{\sigma }_{{A}_{Y}}^{2}\right)+{\sigma }_{U}^{2}+{\sigma }_{E}^{2}}$.    

### Software

We used the R-software [36] for data handling. We estimated variance components and predicted breeding values with the DMU software [37]. Variance components were estimated with the Average-Information Restricted Maximum Likelihood (AI-REML) algorithm in DMUAI [38]. For the relative risk models, breeding values were predicted with the Jacobi Conjugate Gradient algorithm and the GLMM module in DMU4 [37]. For the linear models, breeding values were predicted with the Preconditioned Conjugate Gradient algorithm in DMU5 [37].

## Results

### Validation criteria

The validation criteria were similar for the single-trait relative risk models and the single-trait linear repeatability models (0.02; Table 2). The validation criteria were larger for the two-trait linear repeatability models than for the single-trait models (0.04 vs. 0.02; Table 2).

**Table 2:** Validation criteria and dispersion coefficients for the different statistical models

Statistical model	Validation criterion	Dispersion coefficient	Accuracy
Single_Relative_GT	0.02^b^	1.21	0.07
Single_Relative_MF	0.02^b^	1.23	0.07
Single_Linear_GT	0.02^b^	0.41	0.07
Single_Linear_MF	0.02^b^	0.42	0.07
Two_Linear_GT	0.04^a^	0.82	0.12
Two_Linear_MF	0.04^a^	0.83	0.12

All validation criteria were significantly different from zero at *P* < 0.05 according to *t*-tests with Bonferroni corrections for 21 tests. ^a−b^Values within a column with different superscripts differ significantly at *P* < 0.05 according to Hotelling-Williams T-squared tests with Bonferroni corrections for 21 tests. Accuracy: the accuracy of predicted breeding values, $cor\left(\mathbf{a},\widehat{\mathbf{a}}\right)$, calculated using that $cor\left(\mathbf{a},\widehat{\mathbf{a}}\right)\approx {\sqrt{{h}^{2}}}^{-1}cor\left(\mathbf{y}_{A},\widehat{\mathbf{a}}\right)$, where $\mathbf{a}$ is a vector of breeding values, $\widehat{\mathbf{a}}$ is a vector of breeding values, and ${h}^{2}$ is the average heritability of $\mathbf{y}_{A}$ for the NoL_GT model

### Dispersion coefficients

For the single-trait models, the dispersion coefficients were closer to one for the relative risk models (1.21–1.23; Table 2) than for the linear repeatability models (0.41–0.42; Table 2). For the linear repeatability models, the dispersion coefficients were closer to one for the two-trait models than for the single-trait models (0.82–0.83 vs. 0.41–0.42; Table 2).

### Heritabilities

The estimates of heritabilities were lower for FtoF, FtoS, and StoF than for NoL (0.01–0.03 vs. 0.08–0.09; Table 3). The estimates of additive genetic variances were generally larger for Landrace than for Yorkshire when estimated with the GT relationship matrices, while the opposite was observed with the MF relationship matrices. The estimates of heritabilities were lower for the StoF phenotype than for the FtoS phenotype (0.01 vs. 0.02–0.03; Table 3).

**Table 3:** Estinates of covariance components and heritabilities

Statistical model	Trait	${\sigma }_{{A}_{L}}^{2}$	${\sigma }_{{A}_{Y}}^{2}$	${\sigma }_{{A}_{S}}^{2}$	${\sigma }_{U}^{2}$	${\sigma }_{E}^{2}$	${h}_{L}^{2}$	${h}_{Y}^{2}$
NoL_GT	NoL	0.48 (± 0.04)	0.43 (± 0.04)	–	0.04 (± 0.00)	4.74 (± 0.03)	0.09 (± 0.01)	0.08 (± 0.01)
Two_Linear_MF^a^	StoF	0.001 (± 0.0000)	0.001 (± 0.0000)	0.000 (± 0.0000)	0.000 (± 0.0000)	0.063 (± 0.0001)	0.01 (± 0.001)	0.01 (± 0.001)
	FtoS	0.003 (± 0.0001)	0.003 (± 0.0001)	0.000 (± 0.0000)	0.001 (± 0.0001)	0.114 (± 0.0003)	0.02 (± 0.001)	0.02 (± 0.001)
Two_Linear_GT	StoF	0.000 (± 0.0001)	0.000 (± 0.0001)	0.001 (± 0.0002)	0.000 (± 0.0000)	0.063 (± 0.0001)	0.01 (± 0.001)	0.01 (± 0.001)
	FtoS	0.004 (± 0.0003)	0.002 (± 0.0002)	0.000 (± 0.0004)	0.001 (± 0.0001)	0.114 (± 0.0003)	0.03 (± 0.002)	0.02 (± 0.002)
Single_Linear_MF^a^	FtoF	0.004 (± 0.0002)	0.004 (± 0.0002)	0.001 (± 0.0000)	0.001 (± 0.0001)	0.148 (± 0.0003)	0.03 (± 0.001)	0.03 (± 0.001)
Single_Linear_GT	FtoF	0.005 (± 0.0004)	0.003 (± 0.0003)	0.000 (± 0.0006)	0.001 (± 0.0001)	0.148 (± 0.0003)	0.03 (± 0.003)	0.02 (± 0.002)
Single_Relative_MF^a^	FtoF	0.026 (± 0.0017)	0.028 (± 0.0018)	0.005 (± 0.0003)	0.012 (± 0.0011)	0.756 (± 0.0016)	0.01 (± 0.001)	0.01 (± 0.001)
Single_Relative_GT	FtoF	0.032 (± 0.0030)	0.024 (± 0.0025)	–	0.012 (± 0.0011)	0.756 (± 0.0016)	0.01 (± 0.001)	0.01 (± 0.001)

^a^Variance components from models with the metafounder relationship matrix were rescaled such that they were comparable to those from models with the partial relationship matrices:

$${\sigma }_{{A}_{Y}}^{2}={\sigma }_{{A}_{MF}}^{2}\left(1-\frac{1}{2}{\gamma }_{Y}\right)$$, $${\sigma }_{{A}_{L}}^{2}={\sigma }_{{A}_{MF}}^{2}\left(1-\frac{1}{2}{\gamma }_{L}\right)$$,

and ${\sigma }_{{A}_{S}}^{2}={\sigma }_{MF}^{2}\frac{1}{8} \left({\gamma }_{L}+{\gamma }_{Y}-2{\gamma }_{LY}\right)$. The breed-specific heriabilities were calculated as ${h}_{L}^{2}=\frac{{\sigma }_{{A}_{L}}^{2}}{{\sigma }_{{A}_{L}}^{2}+{\sigma }_{U}^{2}+{\sigma }_{E}^{2}}$ for Landrace

and ${h}_{Y}^{2}=\frac{{\sigma }_{{A}_{Y}}^{2}}{{\sigma }_{{A}_{Y}}^{2}+{\sigma }_{U}^{2}+{\sigma }_{E}^{2}}$ for Yorkshire

### Genetic correlations

The estimates of genetic correlations between breed-specific effects for StoF and FtoS were moderate to high (0.55–0.65; Table 4). The estimated genetic correlations between these effects were larger for the Two_Linear_GT model than for the Two_Linear_MF model (0.64–0.65 vs. 0.55). The standard error of the estimate of the genetic correlation between segregation effects for StoF and FtoS was larger than the estimate of the genetic correlation for these effects (0.51 vs. − 0.37; Table 4).

**Table 4:** Estimates of genetic correlations between StoF and FtoS

Statistical model	Landrace	Yorkshire	Segregation
Two_Linear_MF	0.55 (± 0.04)	0.55 (± 0.04)	0.55 (± 0.04)
Two_Linear_GT	0.64 (± 0.07)	0.65 (± 0.08)	− 0.37 (± 0.51)

Landrace: The additive genetic correlation between breed-specific effects for Landrace. Yorkshire: The additive genetic correlation between breed-specific effects for Yorkshire. Segregation: The additive genetic correlation between segregation effects for crossbreds between Landrace and Yorkshire

## Discussion

In this study, the single-trait relative risk models did not perform better based on the validation criterion than the single-trait linear repeatability models, while the two-trait linear repeatability models performed better than the single-trait linear repeatability models. Thus, the two-trait linear repeatability model seems the most appropriate model for sow survival in this population.

Our estimates of validation criterion and accuracy were low for all models (Table 2). This shows that there is much room for improvement of the statistical models when the aim is prediction of breeding values for making selection decisions. However, the aim of this study was to rank statistical models based on their ability to support prediction. In the following, we argue that the validation criterion we used is suitable for our aim, while also acknowledging both that the two-trait linear model needs to be explored in other populations to confirm its advantage, and that the two-trait model should be improved upon before it is used for selection decisions.

### Small values for the validation criterion and accuracy

The absolute values for the validation criterion were small for all models (0.02–0.04). They were likely small because the accuracies of predicted breeding values, $cor\left({\mathbf{a}},\widehat{{\mathbf{a}}}\right)$, were small and the heritability of NoL was relatively small. In turn, the accuracies of the predicted breeding values were small because: (1) the heritability was small, (2) the breeding values were predicted with pedigree information only, and (3) validation animals likely have few close relatives with complete survival times because they are obtained late in life, only female relatives can obtain phenotypes, female half-sibs of the validation animals were born around the same time as the validation animals and were most likely still alive, and because the mothers and aunts of the validation animals may also have been alive. However, although the prediction models are not accurate for the prediction of adjusted phenotypes, we were not interested in adjusted phenotypes but in the unobservable breeding values, and large heritabilities and accuracies of predicted breeding values are beneficial, but not necessary, to test which of the prediction models predicted the most accurate breeding values.

To our knowledge, Iversen et al. [7] is currently the most comprehensive study on prediction of breeding values for survival of crossbred sows in commercial herds. They predicted breeding values for StoF in time period 1 (they call it LGY12), FtoF (they call it Survival), and NoL between the first and fifth parity (they call it LGY15) among purebred animals of breeds A or B, their crossbreds, and combinations of datasets for purebred and crossbred animals. Among their results, we focus on their ability to predict breeding values for LGY15 among crossbred animals using a model for Survival because that is both most similar to our approach and what they recommended. They estimated accuracies of predicted breeding values of 0.003 when using crossbred data only, 0.015 when using both crossbred data and data from breed A, 0.112 when using both crossbred data and data from breed B, and 0.038 when using crossbred data and data from breeds A and B. Hence, most of their estimated accuracies of predicted breeding values were similar to those estimated here (0.07–0.12, Table 2). However, they used three approaches that are expected to increase the accuracy of predicted breeding values compared to our study. First, they predicted breeding values using genomic information rather than pedigree information only, and genomic information is generally most beneficial for traits with low heritabilities, including sow survival [38]. Second, they used purebred survival as an indicator trait for commercial sow survival. This increased the accuracy of predicted breeding values, because their genetic correlations of survival between purebreds and crossbreds were estimated to be positive (0.25–0.61), their heritability estimate for LGY15 was larger for purebred than for crossbred animals, and because they had fewer observations on crossbred than on purebred animals (Breed A: 19,320; Breed B: 128,843; Crossbred: 12,079 observations). Lastly, they constructed training and validation datasets based on birth dates rather than observation dates. However, in practice, breeders can only predict breeding values with information obtained prior to a cut-off date (as emulated in this study). Our accuracies of predicted breeding values are nevertheless on par with those from Iversen et al. [7], although we used a more realistic and stricter testing scheme, no genomic information, and no indicator traits. Therefore, we see this study as an improvement upon existing literature on prediction of breeding values for sow survival.

Larger accuracies of predicted breeding values could have been obtained in several ways. For example, we could have predicted breeding values using the single-step procedure rather than with pedigree information only. However, we see no reason to believe that genomic information changes the ranking between the prediction models. We could also have included indicator traits in the prediction models to support the prediction of breeding values for sow survival. However, indicator traits should be sought after a prediction model has been established for the focal trait, which was the focus of this study. Furthermore, indicator traits that are relevant for prediction of breeding values in commercial sows are not necessarily relevant for prediction of breeding values of selection candidates. Therefore, we saw no reason to implement measures that increased the accuracies of predicted breeding values.

### Linear repeatability vs. relative risk models

We expected that the relative risk models would predict breeding values for sow survival more accurately than the linear repeatability models but we found that they were equally accurate. Therefore, instead of confirming our hypothesis, this study expands on the collection of studies that have found the predictive performances of linear models for binary survival traits to be comparable to those of non-linear models, such as probit models and logit models [26, 33, 39]. Compared to the latter studies, we also used generalized linear models with the logarithm link function and the Poisson variance function, and our linear repeatability models differs from the commonly used linear models for survival traits [18, 26, 39] because the linear repeatability models assume that the survival trait consists of multiple partial survival traits rather than survival across one time period [19]. This poses additional challenges for the linear repeatability models, because they need to accurately combine information on survival across multiple time periods, although survival rates differ across time periods [20]. Nevertheless, linear repeatability models seem preferable over relative risk models for implementation into practical breeding goals, because they provide similar prediction accuracies, are easier to develop, easier to combine into multi-trait models, and computationally less demanding.

### Two-trait vs. single-trait models

The greater accuracy we observed for two- compared to single-trait models was mainly the result of the following three mechanisms. First, the two-trait models can account for differences in both environmental and genetic effects between the two stages in the reproductive cycle, which should lead to higher prediction accuracy [40]. Second, the two-trait models can incorporate differences in variance components that arise from the differences in mortality rates between the two stages of the reproductive cycle (0.12–0.17 vs. 0.07; Table 1) [4, 5]. Third, the two-trait models predicted breeding values with many more phenotypic records than the single-trait models (StoF: 244,331 + FtoS: 207,225 vs. FtoF: 171,110; Table 1) because they include phenotypes for survival from the first service to the first farrowing (time period 0 for StoF; Fig. 2), and because it takes fewer days to decide whether sows are dead when such information is inferred rather than observed as it was in this study (121 and 70 days vs. 190 days, see Additional File 2). As a result, the two-trait models have access to more phenotypes from both young and older animals that are alive compared to the single-trait models. Furthermore, the additional phenotypes from young animals are likely much more informative for the prediction of breeding values of validation animals than older records, because animals that were serviced just before the validation animals are likely more closely related to validation animals than older animals – e.g., as full-sibs, half-sibs, or cousins. In conclusion, the two-trait models likely predict more accurate breeding values for sow survival because they more accurately fit the data and because they have access to more phenotypes.

### Multibreed relationship matrices

We used two types of multibreed relationship matrices to examine whether our conclusions depended on the choice between them. The application of multibreed relationship matrices to crossbred data is still quite understudied and some results are conflicting; Legarra et al. [11] stated that the two relationship matrices we used are equivalent under certain conditions; Poulsen et al. [13] confirmed near-equivalence in small simulated crossbred populations with no selection and simple phenotypes; Mei et al. [28] found that the MF relationship matrices generally resulted in slightly more accurate predictions than the GT relationship matrices but the ranking between the multibreed relationship matrices depended on the trait; and we found no effect on validation criterion of choosing between the multibreed relationship matrices.

Despite having no effect on validation criterion, the multibreed relationship matrices affect the estimated variance components (Table 3). In general, the breed-specific variances were similar or larger for Landrace than for Yorkshire when the model used GT relationship matrices, while the opposite was observed for models with the MF relationship matrix, and the segregations variances were smaller for the models with the GT relationship matrices compared to those from models with the MF relationship matrix. Consequently, in this study, the different multibreed relationship matrices affected how the genetic variance was partitioned into the breed-specific variances that is eligible for purebred selection, and the segregation variance that only arise in crossbred individuals. Since the estimates of breed-specific variances differ, the expected ability to select for sow survival in each breed also differs. Therefore, further studies are needed to investigate how the choice of multibreed relationship matrix affects the prediction of breeding values among both crossbred animals with phenotypes and selection candidates.

### Definition of sow survival in discrete rather than continuous time

We defined phenotypes for sow survival in discrete time. One reason was that we aimed to predict breeding values for survival to the next farrowing [21] because sows generate revenue when they farrow rather than per day. Another reason was that we expect the effects of many mortality risks to operate per reproductive cycle rather than per day. For example, the risk of culling after farrowing is generally not larger for sows with longer lactations [41]. Finally, we only observed when sows were serviced and farrowed, and not when they died. Thus, we were not able to determine the exact number of days sows were alive or in production. Therefore, defining sow survival in discrete time rather than in continuous time was more appropriate.

### Validation of statistical models

The validation criterion used in this study was not calculated as in many other studies. First, we validated the predicted breeding values using a different trait than they were predicted with. Second, we transformed the predicted breeding value to a different scale. Lastly, we defined a different time period for sow survival than other studies.

We chose to validate the predicted breeding values using adjusted phenotypes for NoL rather than adjusted phenotypes for sow survival because of the arguments mentioned in the Validation paragraphs of the Methods section, i.e., sow survival only has repeated observations for the phenotypically best animals, and the validation on a single time period is inaccurate because sow survival is a genetically complex trait [20]. This also allowed two- and single-trait models to be compared, despite they utilized different definitions for time periods for sow survival. A possible alternative would have been to first estimate correlations between predicted breeding values and adjusted phenotypes for survival in each time period of interest and then combining this information into one validation criterion. However, it is not clear how such an approach can account for the fact that sows that are alive in later time periods generally have higher breeding values for sow survival because of phenotypic selection across time periods, and that time periods are not of equal importance due to differences in survival rates and numbers of animals at risk. Another benefit of using adjusted phenotypes for NoL rather than sow survival to compute the validation criterion is that it resulted in greater statistical power for testing differences between accuracies, because the heritability of NoL is larger than that of survival [7, 9, 20, 32]. One downside is that the predicted breeding values also need to be transformed if the dispersion coefficients are of interest [see Additional File 3]. Dispersion coefficients on untransformed predicted breeding values could, however, also be estimated using the LR approach [42]. In principle, the validation criterion for accuracy can also be estimated using the LR approach, but this approach relies on a high level of accordance between the data and the statistical model – which is not needed for the validation criterion used in this study. Therefore, to the best of our knowledge, the most appropriate approach for comparing predictive performances for sow survival is to use adjusted phenotypes for a longevity trait such as NoL, while the dispersion coefficient can be calculated in other ways than what we used.

We defined NoL differently than other studies as we included survival between the first service and the first farrowing, while other studies on sow survival exclude this time period [7, 27, 43]. One argument for omitting this time period is that only a small fraction of sows at risk is removed during this time period. Although this is true [20], more sows are at risk during this period [20]. In Danish commercial herds, 7% of sows that have been serviced are removed during this period, while on average, 12% of these sows are removed during the following four reproductive cycles [20]. Therefore, we argue that the time period between the first service and the first farrowing is important and should be included in the phenotype for selection.

### Perspectives

Sow survival is an economically and societally important trait with many opportunities to improve prediction of breeding values. First, sow survival should be measured in commercial sows [9]. Nevertheless, most genetic analyses have been on survival of purebred sows in nucleus or multiplier herds [21, 24, 44–49], and only few studies have analyzed sow survival in commercial herds [7, 9, 20, 43]. Second, the crossbred genetic background of commercial sows should be appropriately accounted for in the relationship matrix [10–13] but only one of the genetic analyses of commercial sow survival has used multibreed relationship matrices [20]. Lastly, commercial sow survival is a genetically complex trait, with a genetic background that changes throughout the life of the sow [20]. Nevertheless, only Engblom et al. [43] and Poulsen et al. [9] have followed sows to later parities –sow survival is generally only followed until the fourth parity [50] or fifth parity [7, 20], at which point 40% of commercial sows are still alive [20]. To our knowledge, our study is the first to combine the use of the current best practices regarding data source, relationship matrix, and lifespan coverage.

## Conclusions

The relative risk and linear repeatability single-trait models for survival between subsequent farrowings predicted equally accurate breeding values (0.02), while the linear repeatability two-trait models for survival from services to their subsequent farrowings and farrowings to the subsequent services predicted more accurate breeding values than the single-trait models (0.04 vs. 0.02). However, the accuracy of breeding values was small for all models because the survival phenotypes used for prediction were censored and the heritability of complete survival times was moderate (8–9%). Therefore, the comparison would benefit from reevaluation in other populations, and the models should be improved upon before implementation in practical breeding programs.

## Supplementary Information

Additional file 1.

Additional file 2.

Additional file 3.

## Data Availability

Data is not available upon request because it is owned by the Danish Agriculture and Food Council F.m.b.a., Axelborg, Axeltorv 3, 1609, Copenhagen V, Denmark.
