# Supplementary material for: Comparison between repeatability, reproductive stage stratified repeatability, and relative risk models for prediction of breeding values for functional survival in rotationally crossbred sows
Source: Genet Sel Evol. 2025 Dec 9;57:72. doi: 10.1186/s12711-025-01019-4 (PMC12715952; doi:10.1186/s12711-025-01019-4)
Supplement: Supplementary file 1 — Additional file 1. [file 12711_2025_1019_MOESM1_ESM.docx]

## Additional file 1: Determining partial survival phenotypes for right-censored animals

We constructed survival phenotypes using service dates and farrowing dates. Consequently, we only observed when sows were alive and not when they were dead, and it was necessary to determine when the absence of service or farrowing records indicated that sows were dead.

We determined that sows had died during a specific time period if the number of days since the last observations was more than the number of days it took for 95% of surviving sows to complete that time period. For FtoF, sows were considered dead if more than 190 days had passed without a farrowing record. For StoF, sows were considered dead when more than 121 days had passed without a farrowing record. For FtoS, sows were regarded as dead when more than 70 days had passed without a service record. The sum of numbers of days for StoF and FtoS was not equal to the number of days for FtoF because they were calculated and rounded to integers separately.

Because of the abovementioned approach, phenotypes from dead sows were generally obtained later than phenotypes from surviving sows. This can affect the prediction of breeding values because contemporary groups, where most sows recently initiated a time period, will seem to have higher survival rates than contemporary groups where all sows have finished their time period. Therefore, we omitted phenotypes for FtoF that were initiated less than 190 days prior to the last date with phenotypes (February 25^th^, 2022), we omitted phenotypes for StoF that were initiated less than 121 prior to the last date with phenotypes, and we omitted phenotypes for FtoS that were initiated less than 70 days prior to the last date with phenotypes.
