# Supplementary material for: Comparison between repeatability, reproductive stage stratified repeatability, and relative risk models for prediction of breeding values for functional survival in rotationally crossbred sows
Source: Genet Sel Evol. 2025 Dec 9;57:72. doi: 10.1186/s12711-025-01019-4 (PMC12715952; doi:10.1186/s12711-025-01019-4)
Supplement: Supplementary file 2 — Additional file 2. [file 12711_2025_1019_MOESM2_ESM.docx]

## Additional file 2: Calculation of the reproductive performance of sows

In the main study, we used the variable *reproductive performance* as a covariate in the linear models for the phenotype *lifetime number of litters produced* (NoL). The challenge with this covariate is that sows do not produce the same number of litters (min: 0; max = 14). This is a challenge because it becomes unclear how to combine multiple litter sizes to one covariate. Furthermore, the number of litters produced is the response variable, and great care must be taken such that the method of calculating the covariate do not create false associations between the covariate and the response variable. Instead, [9] proposed to define reproductive performance as the permanent environmental effect from a linear mixed model. This approach directly shrinks litter size observations from sows that have produced few litters, while still providing a single covariate.

The model that we used to calculate the reproductive performances was: $\begin{aligned} \mathbf{y}=\mathbf{Xb}+\mathbf{Wg}+\mathbf{e} ,\#\#\left( SEQ Equation \backslash* ARABIC 21 \right) \end{aligned}$

where **y** is a vector of litter sizes (with potentially multiple observations per sow); **b** is a vector of fixed parameters for herd-year-month at birth and parity; **g** is a vector with random permanent sow effects; **e** is a vector of residuals; and **X** and **W** are design matrices. The vector of predicted permanent sow effects was centered to obtain a vector of reproductive performances with an average equal to 0. Some animals did not obtain reproductive performance through Eq. $21$ because they were culled as gilts. The reproductive performance of these animals was assumed to be zero and was not included during the centering – i.e., the reproductive performance of animals culled as gilts was equal to the average reproductive performance. The vector of reproductive performances was predicted using the dataset for validating predicted breeding values.
