# Supplementary material for: Comparison between repeatability, reproductive stage stratified repeatability, and relative risk models for prediction of breeding values for functional survival in rotationally crossbred sows
Source: Genet Sel Evol. 2025 Dec 9;57:72. doi: 10.1186/s12711-025-01019-4 (PMC12715952; doi:10.1186/s12711-025-01019-4)
Supplement: Supplementary file 3 — Additional file 3. [file 12711_2025_1019_MOESM3_ESM.docx]

## Additional file 3: Validation of breeding values for repeated survival traits

### Background

In the main article, we validated predicted breeding values for sow survival by first transforming them to the *number of litters produced* (NoL) scale and then calculating their correlation with adjusted phenotypes for NoL. This is not a standard approach, as predicted breeding values generally are validated with the trait definition they are predicted with. In the following, we motivate our approach using results from a simple simulation study and the premise that survival is the process of attaining longevity.

The aim of this small simulation study was to find a combination of scales for predicted breeding values and adjusted phenotypes where the correlation is representative of the accuracy of the breeding value, the regression coefficient is approximately one for the regression of adjusted phenotypes onto predicted breeding values, and where the statistical for testing the difference between two correlations is larger.

### Simulation

First, we initialized 1,000 sows with breeding values, $\boldsymbol{a\sim}\mathcal{N}\left( \begin{matrix} \boldsymbol{0}, & \boldsymbol{I}\sigma_{A}^{2} \end{matrix} \right)$, and predicted breeding values with 51 different accuracies, ${\hat{\boldsymbol{a}}}_{r}\mathcal{\sim N}\left( \begin{matrix} c_{r}^{2}\boldsymbol{a}, & \left( c_{r}^{2}-c_{r}^{4} \right)\sigma_{A}^{2}\boldsymbol{I} \end{matrix} \right)$, where subscript $r\in\left\{ 1,2,\ldots,51 \right\}$ denotes the simulated accuracy of breeding values, $\mathcal{N}\left( \ldots\right)$ denotes the normal distribution, $\sigma_{A}^{2}=0.004$ is the additive genetic variance that we estimated for Landrace with the Single_Linear_MF model in the main article, $\boldsymbol{c}=\left\{ 0,0.02,0.04,\ldots,1 \right\}$ is a vector of accuracies for predicted breeding values, $\boldsymbol{a}$ is a vector of breeding values, and $\boldsymbol{I}$ is an identity matrix. Then, we simulated the survival of these sows using the following average survival rates during time periods: $\boldsymbol{\mu=}\left\{ \begin{matrix} \begin{matrix} 0.93 & 0.85 & 0.85 \end{matrix} & \begin{matrix} 0.81 & 0.74 & 0.60 \end{matrix} & \begin{matrix} 0.60 & \ldots& 0.60 \end{matrix} \end{matrix} \right\}$, where the first five survival rates were recently reported survival rates to farrowing from the same population as in the main article [1]. In the simulation, the survival phenotypes were sequentially sampled as:

$$\begin{aligned} \boldsymbol{y}_{j}=\left\{ \begin{aligned} Bernoulli\left( \mu_{j}\boldsymbol{1}+\boldsymbol{a} \right), &j\leq15& \\ \boldsymbol{0}, &otherwise \end{aligned} \right.,\#\left( SEQ Equation \backslash* ARABIC 22 \right) \end{aligned}$$

where $\boldsymbol{y}_{j}$ is the survival phenotype where ones denoted survival and zeros denoted death; $Bernoulli(\ldots)$ is the Bernoulli distribution; $j\in\left\{ 1,2,\ldots,16 \right\}$ denotes the time period; $\mu_{j}$ is the average survival rate; and $\boldsymbol{a}$ is the breeding value. The elements of $\boldsymbol{y}_{j}$ were restricted such that their bounds were $\left[ 0,1 \right]$ by shrinking values above one to one and increasing value below zero to zero. For each time period, $j$, we only simulated phenotypes for sows that were alive at the previous time period, $j-1$. The simulation was replicated 1000 times.

After the simulation, we defined two types of predicted breeding values: predicted breeding values for survival as simulated above (${\hat{\boldsymbol{a}}}_{Surv}$) and predicted breeding values for *lifetime* *number of time periods survived* (longevity) as calculated in the Validation section of the main article (${\hat{\boldsymbol{a}}}_{Long}$). We also defined five adjusted phenotypes: repeated survival across all time periods adjusted for the average survival rate across all time periods ($\boldsymbol{y}_{Survall}$), repeated survival across all time periods adjusted for time period-specific average survival rates ($\boldsymbol{y}_{Survallc}$), survival to the first time period ($\boldsymbol{y}_{Surv01}$), survival from the second to the third time period ($\boldsymbol{y}_{Surv23}$), and longevity ($\boldsymbol{y}_{Long}$). The vectors $\boldsymbol{y}_{Survall}$ and $\boldsymbol{y}_{Survallc}$ are repeated traits, so when those traits were analyzed, the elements in vectors of breeding values were repeated to account for this.

### Validation

The first desired characteristic was that the predictive performance, $cor\left( \boldsymbol{y}_{A},\hat{\boldsymbol{a}} \right)$, was strongly indicative of the accuracy of the predicted breeding values, $cor\left( \boldsymbol{a},\hat{\boldsymbol{a}} \right)$, such that $\left( \sqrt{h^{2}} \right)^{-1}cor\left( \boldsymbol{y}_{A},\hat{\boldsymbol{a}} \right)\approx cor\left( \boldsymbol{a},\hat{\boldsymbol{a}} \right)$. This was examined using a linear model:

$\begin{aligned} y_{r}=\mu_{1}\boldsymbol{+}\beta_{1}x_{r}+e_{r},\#\left( SEQ Equation \backslash* ARABIC 23 \right) \end{aligned}$where $y_{r}$ is the predictive performance, $\left( \sqrt{h^{2}} \right)^{-1}cor\left( \boldsymbol{y} ,{\hat{\boldsymbol{a}}}_{r} \right)$; $x_{r}$ is the true accuracy of breeding values, $cor\left( \boldsymbol{a},{\hat{\boldsymbol{a}}}_{r} \right)$; subscript $r\in\left\{ 1,2,\ldots,51 \right\}$ denotes a simulated prediction accuracy; $\beta_{1}$ is a regression coefficient; $\mu_{1}$ is an intercept; $e_{r}\sim\mathcal{N}\left( 0,\sigma_{E1}^{2} \right)$ is a residual; and $\sqrt{h^{2}}$ is the square root of the heritability of adjusted phenotypes estimated as $cor\left( \boldsymbol{y} ,\boldsymbol{a} \right)$ because $cor\left( \boldsymbol{y} ,\boldsymbol{a} \right)=\frac{\sigma_{y,a}}{\sqrt{\sigma_{y}^{2}\sigma_{a}^{2}}}=\frac{\sigma_{a}^{2}}{\sqrt{\sigma_{y}^{2}\sigma_{a}^{2}}}=\sqrt{\frac{\sigma_{a}^{2}}{\sigma_{y}^{2}}}=\sqrt{h^{2}}$. The model in Eq. 23 was estimated separately for each combination of adjusted phenotype, type of predicted breeding value, and replicate. We regarded the relationship as satisfactory if the average values of $\mu_{1}$, $\beta_{1}$, and $\sigma_{E1}^{2}$ were close to 0, 1, and 0, respectively.

The second desired characteristic was that the linear regression coefficient of adjusted phenotypes onto predicted breeding values was approximately one. This was also examined using a linear model:

$$\begin{aligned} y_{ri}=\mu_{2}\boldsymbol{+}\beta_{2}\hat{a}_{ri}+e_{ri},\#\left( SEQ Equation \backslash* ARABIC 24 \right) \end{aligned}$$

where $y_{ri}$ is an adjusted phenotype, $\hat{a}_{ri}$ is a predicted breeding value, $\mu_{2}$ is a general mean, $\beta_{2}$ is a regression coefficient, and $e_{ri}\sim\mathcal{N}\left( 0,\sigma_{E2}^{2} \right)$ is a residual. The model was estimated separately for each combination of adjusted phenotype, type of predicted breeding value, and replicate. We regarded the relationship as satisfactory if the average value of $\beta_{2}$ was approximately one.

The third desired characteristic was that the statistical power for testing the difference between two predictive performances is large. The statistical power is large, when the absolute difference between two correlations is large [2]. Therefore, given that the first desired characteristic is upheld, the statistical power is large when the heritability is large:

$$\begin{aligned} \begin{matrix} \Delta cor\left( \boldsymbol{y}_{A},\hat{\boldsymbol{a}} \right) & = & cor\left( \boldsymbol{y}_{A},{\hat{\boldsymbol{a}}}_{1} \right)-cor\left( \boldsymbol{y}_{A},{\hat{\boldsymbol{a}}}_{2} \right) \\ & \approx& \sqrt{h^{2}}\left( cor\left( \boldsymbol{a},{\hat{\boldsymbol{a}}}_{1} \right)-cor\left( \boldsymbol{a},{\hat{\boldsymbol{a}}}_{2} \right) \right) \\ & \approx& cor\left( \boldsymbol{y}_{A},\boldsymbol{a} \right)\left( cor\left( \boldsymbol{a},{\hat{\boldsymbol{a}}}_{1} \right)-cor\left( \boldsymbol{a},{\hat{\boldsymbol{a}}}_{2} \right) \right) \end{matrix},\#\left( SEQ Equation \backslash* ARABIC 25 \right) \end{aligned}$$

where everything is as previously described, and $cor\left( \boldsymbol{a},{\hat{\boldsymbol{a}}}_{1} \right)-cor\left( \boldsymbol{a},{\hat{\boldsymbol{a}}}_{2} \right)$ is constant across types of adjusted phenotypes. Therefore, the average correlation between adjusted phenotypes and breeding values across simulations, $\bar{cor\left( \boldsymbol{y}_{A},\boldsymbol{a} \right)}$, indicates the relationship between statistical powers of the approaches.

### Results

For the first desired characteristic, $\left( \sqrt{h^{2}} \right)^{-1}cor\left( \boldsymbol{y}_{A},\hat{\boldsymbol{a}} \right)\approx cor\left( \boldsymbol{a},\hat{\boldsymbol{a}} \right)$, there was a strong and linear relationship between most combinations of adjusted phenotypes and predicted breeding values as the average values of $\mu_{1}$ ranged from 0.02 to 0.08, $\beta_{1}$ ranged from 0.90 to 0.97, and $\sigma_{E1}^{2}$ ranged from 0.06 to 0.19 (Table 5). The exception was for the corrected phenotype $\boldsymbol{y}_{Surv23}$ where $\mu_{1}$ ranged from -0.27 to -0.21, $\beta_{1}$ ranged from 1.14 to 1.17, and $\sigma_{E1}^{2}$ ranged from 5.62 to 24.1.

For the second desired characteristic, the regression coefficient for the regression of adjusted phenotypes onto predicted breeding values, $\beta_{2}$, was highly affected by the combinations of adjusted phenotypes and predicted breeding values (0.03-16.2, Table 5). However, the average regression coefficients were approximately one for the regressions of $\boldsymbol{y}_{Long}$ onto ${\hat{\boldsymbol{a}}}_{Long}$ (1.06) and $\boldsymbol{y}_{Surv01}$ onto ${\hat{\boldsymbol{a}}}_{Surv}$ (1.18) – that is, when the adjusted phenotypes and predicted breeding values are on the same scale, at most one time period is considered, and that time period is the first time period.

For the third desired characteristic, there were considerable differences between the average correlation between adjusted phenotypes and breeding values (0.07-0.37, Table 5) which shows that the statistical power differs between the approaches. The largest values were 0.37 for $\boldsymbol{y}_{Long}$, 0.20-0.21 for $\boldsymbol{y}_{Surv01}$, and 0.18 for $\boldsymbol{y}_{Surv23}$.

Table 5: Summary statistics (mean ± sd) across replicates

| $\boldsymbol{y}_{\boldsymbol{A}}$ | $\hat{\boldsymbol{a}}$ | $\bar{\sqrt{\boldsymbol{h}^{\boldsymbol{2}}}}$ | $\boldsymbol{\mu}_{\mathbf{1}}$ | $\boldsymbol{\beta}_{\mathbf{1}}$ | $\boldsymbol{\beta}_{\mathbf{2}}$ | $\boldsymbol{\sigma}_{\boldsymbol{E}\mathbf{1}}^{\mathbf{2}}$ |
| --- | --- | --- | --- | --- | --- | --- |
| $\boldsymbol{y}_{\boldsymbol{Long}}$ | ${\hat{\boldsymbol{a}}}_{Surv}$ | 0.37 | 0.02±0.08 | 0.97±0.12 | 16.2±34.4 | 0.06±0.04 |
| $\boldsymbol{y}_{\boldsymbol{Long}}$ | ${\hat{\boldsymbol{a}}}_{Long}$ | 0.37 | 0.02±0.08 | 0.97±0.12 | 1.06±2.26 | 0.06±0.05 |
| $\boldsymbol{y}_{\boldsymbol{Survall}}$ | ${\hat{\boldsymbol{a}}}_{Surv}$ | 0.07 | 0.02±0.08 | 0.97±0.13 | 0.44±0.92 | 0.06±0.04 |
| $\boldsymbol{y}_{\boldsymbol{Survall}}$ | ${\hat{\boldsymbol{a}}}_{Long}$ | 0.07 | 0.02±0.08 | 0.97±0.12 | 0.03±0.06 | 0.06±0.05 |
| $\boldsymbol{y}_{\boldsymbol{Survallc}}$ | ${\hat{\boldsymbol{a}}}_{Surv}$ | 0.14 | 0.02±0.08 | 0.97±0.12 | 0.75±1.79 | 0.08±0.06 |
| $\boldsymbol{y}_{\boldsymbol{Survallc}}$ | ${\hat{\boldsymbol{a}}}_{Long}$ | 0.14 | 0.02±0.08 | 0.97±0.12 | 0.05±0.12 | 0.08±0.08 |
| $\boldsymbol{y}_{\boldsymbol{Surv}\mathbf{01}}$ | ${\hat{\boldsymbol{a}}}_{Surv}$ | 0.21 | 0.08±0.16 | 0.90±0.24 | 1.18±3.48 | 0.19±0.17 |
| $\boldsymbol{y}_{\boldsymbol{Surv}\mathbf{01}}$ | ${\hat{\boldsymbol{a}}}_{Long}$ | 0.20 | 0.08±0.15 | 0.90±0.23 | 0.08±0.23 | 0.17±0.13 |
| $\boldsymbol{y}_{\boldsymbol{Surv}\mathbf{23}}$ | ${\hat{\boldsymbol{a}}}_{Surv}$ | 0.18 | -0.21±0.27 | 1.14±0.37 | 0.44±7.46 | 5.62±13.6 |
| $\boldsymbol{y}_{\boldsymbol{Surv}\mathbf{23}}$ | ${\hat{\boldsymbol{a}}}_{Long}$ | 0.18 | -0.27±0.38 | 1.17±0.47 | 0.03±0.49 | 24.1±68.6 |

Altogether, we found that: 1) all combinations of adjusted phenotypes and types of predicted breeding values can estimate the accuracy of predicted breeding values given that the scaling factor can be estimated; 2) the regression coefficient for adjusted phenotypes onto predicted breeding values can be expected to be around one when they are on the same scale, at most one time period is considered and that time period is the first time period; and 3) the statistical power for comparing predictive performances is largest for adjusted phenotypes for longevity. Therefore, this motivates our approach in the main article, where we used adjusted phenotypes for longevity for validation of predictive abilities for repeated survival traits with phenotypic selection across time periods.

### References

[1] Poulsen BG, Leite NG, Lourenco D. Genetic associations between survival at different parities in commercial rotationally crossbred sows. In: Proceedings of the 12th World Congress on Genetics Applied to Livestock Production: 3-8 July 2022; Rotterdam. 2022.

[2] Dunn OJ, Clark V. Comparison of Tests of the Equality of Dependent Correlation Coefficients. J Am Stat Assoc. 1971;66:904-8.
